# Overexpression of the grapevine PGIP1 in tobacco results in compositional changes in the leaf arabinoxyloglucan network in the absence of fungal infection

**DOI:** 10.1186/1471-2229-13-46

**Published:** 2013-03-18

**Authors:** Eric Nguema-Ona, John P Moore, Alexandra D Fagerström, Jonatan U Fangel, William GT Willats, Annatjie Hugo, Melané A Vivier

**Affiliations:** 1 Institute for Wine Biotechnology, Department of Viticulture and Oenology, Faculty of AgriSciences, Stellenbosch University, Matieland, 7602, South Africa; 2 Energy Biosciences Institute, University of California, 2151 Berkeley Way, Berkeley, CA, 94720-5230, USA; 3 Department of Plant Biology and Biotechnology, Faculty of Life Sciences, University of Copenhagen, DK-, 1001, Denmark; 4 Department of Microbiology, Stellenbosch University, Matieland, 7602, South Africa; 5 Current address: Laboratoire Glycobiologie et Matrice Extracellulaire Végétale (Glyco-MEV). Grand Réseau de Recherche VASI de Haute Normandie, PRES Normandie Université. Université de Rouen, Mont Saint Aignan, 76821 Cedex, France

**Keywords:** Tobacco, Grapevine, Cell wall, Profiling, VvPGIP1, Polygalacturonase-inhibiting protein (PGIP), Endopolygalacturonase (ePG), Arabinoxyloglucan (AXyG), Xyloglucan-specific endoglucanase (XEG), Arabinogalactan proteins (AGPs)

## Abstract

**Background:**

Constitutive expression of *Vitis vinifera* polygalacturonase-inhibiting protein 1 (*Vvpgip1*) has been shown to protect tobacco plants against *Botrytis cinerea*. Evidence points to additional roles for VvPGIP1, beyond the classical endopolygalacturonase (ePG) inhibition mechanism, in providing protection against fungal infection. Gene expression and biochemical datasets previously obtained, in the absence of infection, point to the cell wall, and particularly the xyloglucan component of transgenic VvPGIP1 lines as playing a role in fungal resistance.

**Results:**

To elucidate the role of wall-associated processes in PGIP-derived resistance pre-infection, a wall profiling analysis, using high-throughput and fractionation techniques, was performed on healthy leaves from wild-type and previously characterized transgenic lines. The cell wall structure profile during development was found to be altered in the transgenic lines assessed versus the wild-type plants. Immunoprofiling revealed subtle changes in pectin and cellulose components and marked changes in the hemicellulose matrix, which showed reduced binding in transgenic leaves of VvPGIP1 expressing plants. Using an enzymatic xyloglucan oligosaccharide fingerprinting technique optimized for tobacco arabinoxyloglucans, we showed that polysaccharides of the XEG-soluble domain were modified in relative abundance for certain oligosaccharide components, although no differences in ion profiles were evident between wild-type and transgenic plants. These changes did not significantly influence plant morphology or normal growth processes compared to wild-type lines.

**Conclusions:**

VvPGIP1 overexpression therefore results in cell wall remodeling and reorganization of the cellulose-xyloglucan network in tobacco in advance of potential infection.

## Background

The cell wall is the first plant compartment that fungal pathogens (necrotrophs or biotrophs) encounter and need to breach for efficient infection to occur [1]. Fungi have evolved a plethora of cell wall degrading enzymes (CWDEs); these include polygalacturonases (PGs), pectin methylesterases (PMEs) and pectate lyases, which are used to attack and colonize potential hosts [2]. *Botrytis cinerea,* is a necrotrophic plant pathogen with a wide host range that infects pectin-rich fleshy fruits through secretion of fungal endopolygalacturonases (ePGs) [3,4] and is responsible for significant crop damage in grapevine (*Vitis vinfera*) in addition to other fruit crops. *Botrytis* releases numerous metabolites and enzymes such as PGs during plant host attack which macerate the pectin component of the cell wall, thus providing the fungi with an entry route and a source of nutrients for growth and proliferation. In response, plants have co-evolved counteracting defence mechanisms, often highly pathogen specific, including an array of cell wall related-responses (for detailed reviews see [5-8]) and also CWDE-inhibiting proteins such as polygalacturonase-inhibiting proteins (PGIPs) and pectin methylesterase-inhibitors (PMEIs) [2]. PGIPs are extracellular leucine-rich repeat (LRR) proteins present in the plant cell wall area with recognition and direct inhibition capabilities towards fungal ePGs [9,10]. Cervone et al. [1] proposed that, during infection, PGIPs decrease the rate of ePG-mediated degradation of homogalacturonan (HG) to oligogalacturonans (OGAs), and thus alter the life span of OGAs, as well as the OGA population’s average molecular length and concentration, which in turn is proposed to elicit a defence response in the infected plant (see also [5,10,11]). Recently, a hybrid kinase consisting of the extracellular domain of *WALL-ASSOCIATED KINASE1* (*WAK1*) and the intra-cellular domain of elongation factor Tu receptor (EFR) kinase was shown to bind OGAs and activate defense responses [12].

The main mechanism-of-action of PGIPs is believed to be through direct inhibition of ePGs [13]. Physical studies have demonstrated direct molecular interactions between PGIPs and ePGs of different origins [14,15]. Until recently, published literature on PGIPs only considered their ePG inhibition functions and most of these studies were conducted *in vitro*. Examples exist however where it has been shown that some PGIP-ePG pairs only interacted in the *in vivo* environment [16], providing strong support that at least some PGIP-ePG pairs require other components to form a functional inhibition complex. It was hypothesized that the binding of PGIP with pectin could be a means to mask the substrate thus protecting it from hydrolysis by ePGs [16-18].

Additional roles outside the normal PGIP-ePG inhibition scenario have been reported recently. Veronico et al. [19] showed that the expression pattern of a native *Pvpgip* could be linked to the degree of resistance of pea plants to cyst nematode infections. Interestingly, no ePG transcripts could be obtained from the cyst nematode, nor was any correlation found between PGIP and native ePG expression, again suggesting that PGIPs could have additional roles in plant-pathogen interactions outside of the classical PGIP-ePG inhibition hypothesis. Similarly, it was found by Kanai et al. [20] that *Atpgip1* transcripts prolonged seed germination by influencing pectin degradation in the seed coat, strongly suggesting that PGIPs probably have multiple functions *in planta*.

By studying VvPGIP1, we have gained significant insight into the *in vivo* functions of PGIPs. Expression analysis of the *Vvpgip1* gene showed that this gene was expressed at the onset of ripening, when fruit cell wall disassembly is known to occur, and berries become more susceptible to pathogen infection [21,22]. *Vvpgip1* was overexpressed constitutively in tobacco (*N. tabacum*) and effective resistance was shown after infection by *B. cinerea* spores by the transformed lines [23]. Transcriptomic and metabolite profiling analysis of these tobacco transgenic lines provided surprising results when the transgenic lines and untransformed controls were compared in the absence of infection (i.e. assessing the baseline changes to the transgenic lines as a consequence of *Vvpgip1* expresssion and without engaging the PGIP-ePG inhibition interaction) [24]. The transgenic lines showed marked changes in their hormone profiles and lignin levels. Moreover, this molecular profiling approach confirmed the importance of the cell wall in PGIP phenotypes and provided the first proof that PGIPs influence cell wall composition and strengthening in a potential priming mechanism [24]. The transcriptomic data obtained, and additional RT-PCR confirmatory experiments, indicated that xyloglucan endotransglycosylase/hydrolase (XET/XTH) genes are down-regulated in *Vvpgip1* transgenic lines compared to wild type plants [24]. XET/XTHs are known to be involved in cell wall xyloglucan remodeling (i.e. tightening and loosening) and hence reorganization of the cellulose/xyloglucan network [25]. Further support for this ‘XET/XTH down-regulation’ data has been obtained showing a similar correlative decrease in total XET enzymatic activity in *Vvpgip1* transformed lines compared to wild-type [24]. These data point to a possible role for the xyloglucan (and possibly the associated cellulose microfibrils) network in the *Vvpgip1* resistance phenotypes observed. In addition, the transcriptomic data showed changes in the expression of CAD (cinnamyl alcohol dehydrogenase) transcripts suggesting modification of lignin metabolism; this was further supported by chemical data which indicated that lignification (and hence wall reinforcement) has occurred in the transgenic lines in the absence of infection [24].

The focus of the work presented here was therefore the cell wall of the well characterized VvPGIP tobacco transgenic lines to investigate the role of the cell wall, and in particular, the arabinoxyloglucan network, in the resistance afforded by *Vvpgip1* expression [16,23,24]. Cell wall profiling methods (including monosaccharide compositional analysis, arabinoxyloglucan oligosaccharide fingerprinting and CoMPP analysis), optimized for tobacco leaves [26] were used. Chemical and enzymatic fractionation was used to thoroughly dissect and compare the arabinoxyloglucan network present in the VvPGIP1 and control material. Developmental profiles, as a function of four leaf positions (i.e. leaf 3 to leaf 6), were obtained from wild type and PGIP overexpressing lines. The arabinoxyloglucan matrix was indeed found to be altered in the *Vvpgip1* tobacco lines. The transformed lines show a modification (versus control plants) in the remodeling and oligomeric composition (e.g. oligosaccharide fingerprint profiles) of the leaf arabinoxyloglucan polymers during development. A model proposing how the overexpression of *Vvpgip1* in tobacco leaves results in a modified cell wall architecture, and oligomeric composition, thus enhancing plant resistance to fungal pathogens both prior to and after infection, is provided.

## Results

### Carbohydrate microarray polymer profiling of SR-1 versus transgenic tobacco lines

Carbohydrate Microarray Polymer Profiling (CoMPP) analysis was used as an initial screening approach to provide an overview of cell wall polysaccharide structures in the plants used in this study. CoMPP analysis was performed on extracted cell wall polysaccharides and allows the profiling of cell wall material using sets of monoclonal antibodies (mAbs) and carbohydrate binding modules (CBMs) which show specificities toward plant cell wall-glycan epitopes [27]. This analysis provides information about the relative abundance of epitopes in the extracted material rather than fully quantitative data, but in contrast to fully quantitative biochemical techniques, it can provide information about polysaccharide rather than monosaccharide occurrence.

CoMPP analysis was performed on alcoholic insoluble residue (AIR), prepared from SR-1 and VvPGIP1 leaf material (for detailed genotypic and phenotypic characterization of transgenic lines 37 and 45, refer to [23,24]). Leaves were harvested when the plants reached the mature 8-leaf stage in order to align the data obtained with whole plant leaf infection assays routinely performed at this same growth state [23]. Four consecutive leaves (labeled from leaf 3 (youngest) to leaf 6 (oldest)) were collected, flash-frozen, and processed to obtain AIR [26].

**Figure 1:**
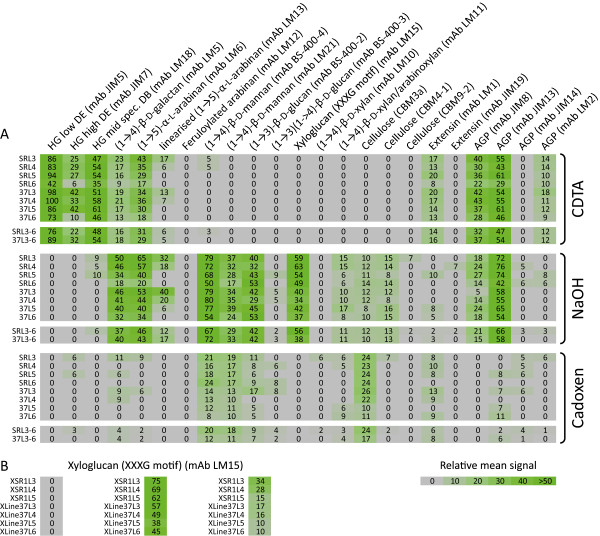
**Comprehensive Microarray Polymer Profiling (CoMPP) analysis of total AIR, fractionated into CDTA, NaOH and Cadoxen subsamples, prepared from individual leaves (leaf 3 to leaf 6) sourced from SR1, 37 and 45 tobacco plants (A).** Analysis of LM15 relative binding to AIR (pre-extracted with sodium acetate buffer), fractionated into CDTA, NaOH and Cadoxen subsamples, prepared from individual leaves (leaf 3 to leaf 6) sourced from SR1, 37 and 45 tobacco plants (**B**). Analyses were performed using four biological replicates for the SR-1 (control) plants and each of the transgenic plants. Two technical replicates were performed for each analysis.

Apart from a few marked differences that will be discussed below, the global CoMPP datasets were similar between wild type and transgenic leaves, suggesting that overall cell wall composition and developmental patterns were not dramatically affected by VvPGIP1 overexpression. Moreover, as the CoMPP pattern of binding was highly similar between lines 37 and 45, only line 37 data is presented in comparison to wild-type (Figure 1). The CoMPP datasets generated confirmed the previous baseline study on *N. tabacum* SR-1 [26] in terms of general composition of the tobacco leaf cell wall. However, the data presented here extends the evaluation of Nguema-Ona et al. [26] by evaluating developmental patterns as a function of leaf position (Figure 1A). A consistent decrease in mAb LM5 binding is seen as a function of leaf age (position 3–6) suggesting galactan turnover during tobacco leaf maturation. Similar stepwise reductions in binding, as a function of leaf position (i.e. from leaf 3 to leaf 6), is evident in the dataset for pectin (i.e. mAbs JIM7 and LM18), arabinans (mAb LM6) and AGPs (mAbs JIM8 and JIM13).

Interestingly, although the developmental trend was generally preserved in the transgenic lines, some epitopes showed differential binding between the WT and transgenic lines (Figure 1A). The most striking feature in the comparative CoMPP data was a marked difference in the binding patterns for mAb LM15 in NaOH extracts. This mAb, raised against xylosylated heptasaccharides from tamarind xyloglucan [28], was found to bind less abundantly to transgenic AIR (VvPGIP1 line 37 and 45) than to that obtained from wild type plants; this difference in binding was found consistently between these samples regardless of leaf position used (Figure 1A). Noticeably, the pattern of differential binding of LM15 remains unchanged when CoMPP analysis was performed on AIR first pre-treated with a sodium acetate buffer extractant (as performed in 26) to remove potentially interfering substances such as pectin and other extracted cell wall proteins (Figure 1B). The influence of wall bound phenolic compounds as interfering compounds in this analysis is possible but probably negligible given the specific differential responses observed in the CoMPP datasets. The xyloglucan epitope recognized by mAb LM15 is likely embedded in the pectin matrix [28], and this step was performed to control for the possibility that pectin and associated buffer soluble components (e.g. cell wall proteins) might block access of the mAb to its target resulting in the differential responses observed for the different populations. Inspection of the dataset obtained after this processing reveals a much more simplified data-matrix (data not shown) than that obtained from total AIR as, for example, most of the AGP epitopes have been removed (data not shown). The data for mAbs LM15 presented in Figure 1 (A and B) strongly support the assertion that either accessibility to, or the abundance of, arabinoxyloglucan polymers has decreased in the transgenic lines tested.

Other changes observed in the transgenic lines included (Figure 1A): (i) The relative amount of binding of mAb JIM7 was enhanced in transgenic material. This might indicate that transgenic plants have elevated levels of pectin esterification versus wild-type; (ii) Similarly, the AGP mAbs JIM8 and JIM13 showed differential binding patterns that could suggest altered AGP organization in the transgenic material; and (iii) subtle differences for CBM3a in Cadoxen extracts between wild-type and transgenic plants was also observed. The reduced binding of mAb CBM3a to transgenic material also suggests that accessibility to the arabinoxyloglucan-cellulose matrix was altered in the VvPGIP1 lines (concordant with the mAb LM15 data).

### Monosaccharide composition analysis of wild type SR-1 versus transgenic tobacco leaves

**Figure 2:**
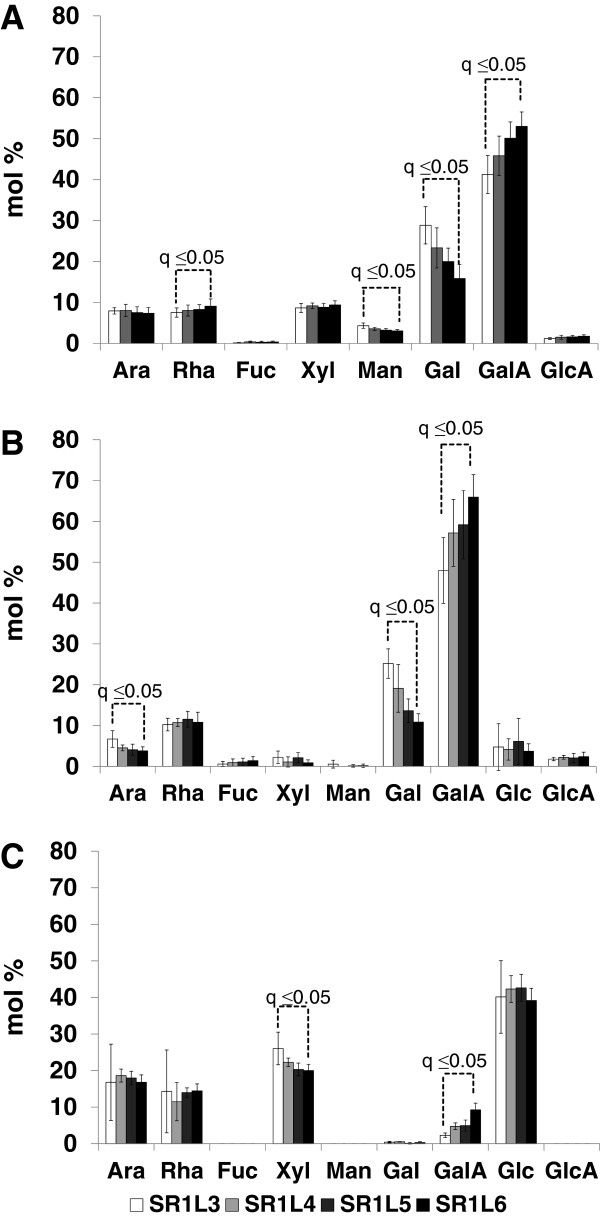
**Evaluation of the leaf developmental cell wall composition profile of SR1 tobacco plants.** Monosaccharide composition analysis of the polymers released after endopolygalacturonase (ePGase) (**A**) and subsequent xyloglucanase (XEGase) treatment of alcohol insoluble residue (AIR) prepared from SR1 tobacco leaves (individual leaves analyzed and labelled leaf 3 to leaf 6 are indicated). Ara: arabinose; Rha: rhamnose; Fuc: fucose; Xyl: xylose; Man: mannose; Gal: galactose; Glu: glucose; GalA: galacturonic acid; GluA: glucuronic acid. Analyses were performed using eight biological replicates for the the SR-1 (control) plants and four biological replicates for each of the transgenic plants. Three technical replicates were performed for each analysis.

To support the CoMPP data other wall profiling methods were used to assess the effect of leaf developmental ‘age’ (i.e. leaf position) on the ‘global’ leaf cell wall composition. The data generated for the SR-1 tobacco population was used as a baseline to compare the transgenic lines. Monosaccharide compositional analysis was used to assess the total cell wall composition (Figure 2A) of AIR generated from leaves 3–6 and revealed Gal and GalA as the main monosaccharides in SR-1, with lesser amounts of Ara, Rha, Xyl and Man, confirming the pectin-rich nature of tobacco leaves (as shown in [26] for leaf position 3). Glc was intentionally removed from the dataset (Figure 2A) as contaminating starch complicated the interpretation. Interestingly, a developmental pattern was evident in the SR-1 data (Figure 2A) where the Gal content decreased stepwise from ca. 25 mol% in leaf 3 to ca. 15 mol% in leaf 6, while a converse staggered increase in GalA abundance from ca. 40 mol% in leaf 3 to ca. 50 mol% was found. Further treatment of the AIR with ePG yielded a soluble fraction (Figure 2B) similar in composition to total AIR except being noticeably deficient in Xyl, Man and Ara. The pattern for Gal and GalA remained the same in the ePG hydrolysed fraction (Figure 2B), although the staggered decreases/increases were from ca. 25 mol% to ca. 10 mol% for Gal and ca. 50 mol% to ca. 70 mol% for GalA (Figure 2A). This data supports the proposal that turnover of galactan chains is occurring in the pectic rhamnogalacturonan I (RG-I) polymer and that this is linked to a shift from actively growing (expanding) leaves to final maturation (see [29,30]). Treatment of the ePG insoluble residue of SR-1 with XEG yielded a soluble fraction rich in Xyl, Glc, Rha and Ara (Figure 2C). A *Paenibacillus sp* xyloglucan specific endoglucanase XEG5 (PspXEG5) was used to digest de-pectinated leaf AIR and the arabinoxyloglucan oligosaccharides released were analyzed using mass spectrometry and high performance anion exchange chromatography (as optimized in [26]). This fraction represents the XEG-soluble fraction domain, one of the three xyloglucan domains present in the wall [31], which is subject to significant metabolic turnover as observed in growing etiolated pea tissue [32,33]. Interestingly, a slight decrease in the Xyl content of this fraction is evident during maturation of the SR-1 leaves (Figure 2C). It is known from literature that tobacco xyloglucan is arabinosylated (i.e. arabinoxyloglucan) and is of the XXGG-type thus possessing a lower degree of xylosyl substitution of the glucan backbone as compared to the XXXG-type present in peas [34]. No significant difference is seen in this fraction (Figure 2C) for the other monosaccharides as a function of leaf position in SR-1 leaves. Furthermore no major differences in global cell wall profiles between wild type and transgenic material was evident for these analyses (Additional file 1 and Additional file 2), confirming our preliminary cell wall compositional analysis of the transgenic lines that showed that VvPGIP1 overexpression did not alter overall cell wall composition [24].

### The leaf arabinoxyloglucan oligomeric composition in SR-1 versus transgenic tobacco lines

**Figure 3:**
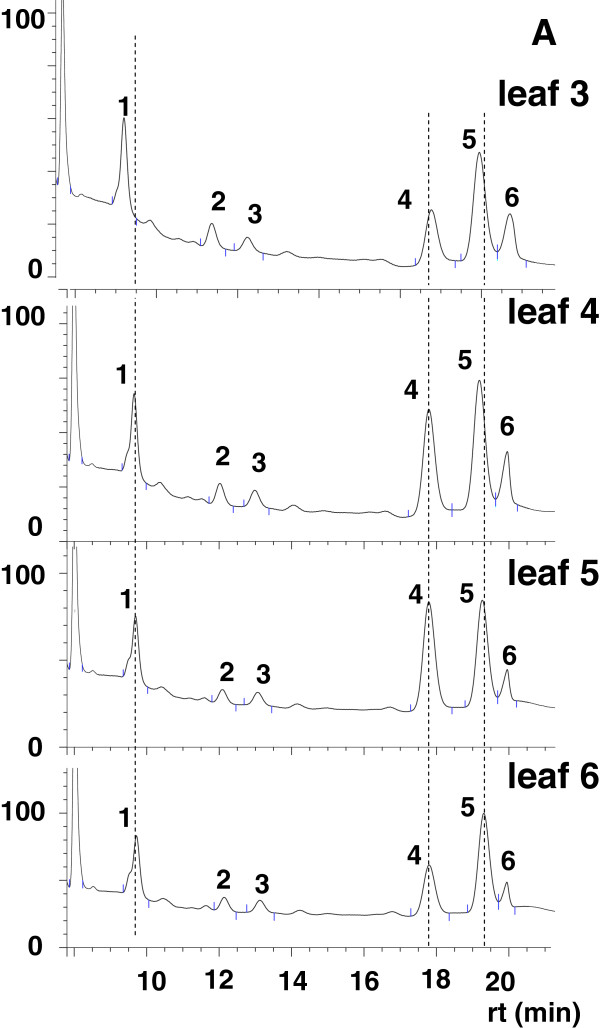
**Evaluation of the leaf developmental arabinoxyloglucan subunit composition profile of SR1 tobacco plants.** A representative trace showing oligomeric digestion products (**A**) released from (de-pectinated) AIR prepared from individual SR1 tobacco leaves (labelled leaf 3 to leaf 6) using liquid ion-exchange chromatography. For a statistical analysis of datasets generated using this approach refer to Figure 4.

**Figure 4:**
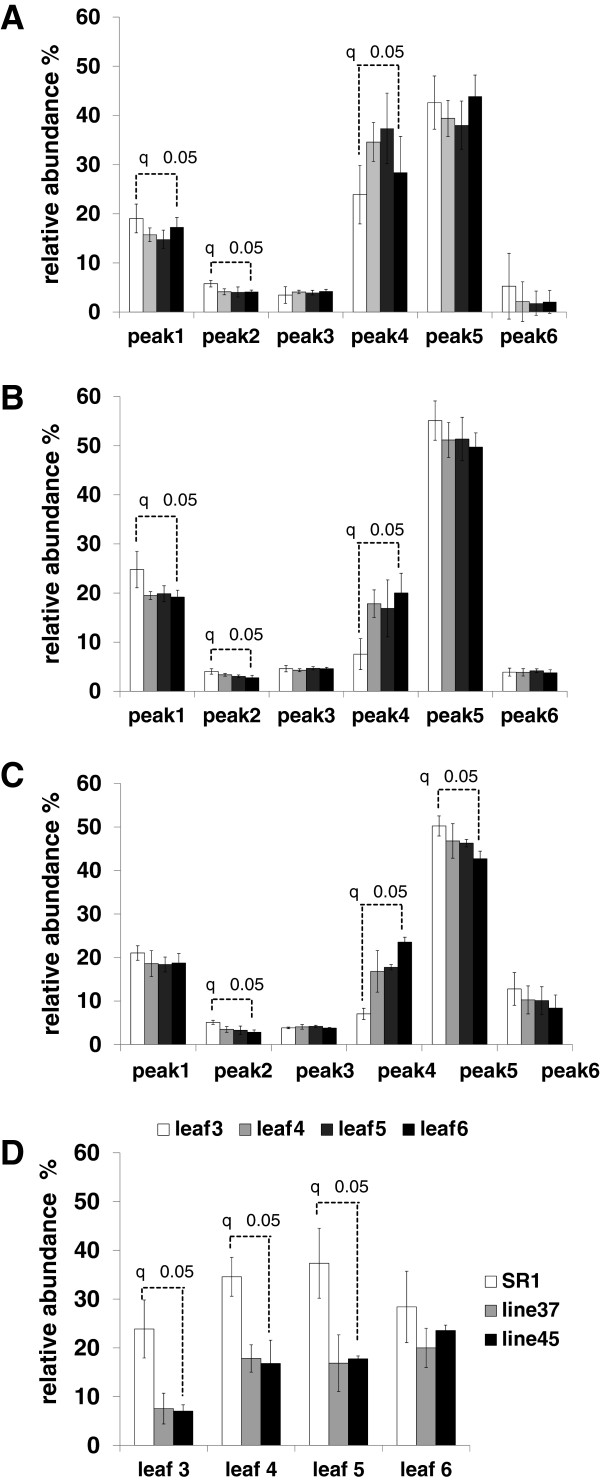
**Relative abundance of peaks labelled 1 to 6 showing a arabinoxyloglucan developmental growth pattern, present in chromatograph traces of oligomeric digestion products of de-pectinated AIR prepared from individual leaves (leaf 3 to leaf 6) sourced from SR1 (A), 37 (B) and 45 (C) tobacco plants.** A comparative graph showing the level of peak 4 as a function of leaf position (leaf 3 to leaf 6) (**D**) in SR1, 37 and 45 tobacco plants is also provided. Analyses were performed using four biological replicates for the the SR-1 (control) plants and each of the transgenic plants. Three technical replicates were performed for each analysis.

Furthermore, a detailed baseline analysis of the leaf arabinoxyloglucan component was conducted on SR-1 plants in preparation for a comparative analysis with the transgenic lines. De-pectinated AIR was isolated from leaves 3–6 and subjected to enzyme oligosaccharide fingerprinting (data presented per leaf position) [35,36]. Enzyme treatment produced six main peaks which were present in all chromatographs generated from each of the four leaf positions assessed (Figure 3). The arabinoxyloglucan oligosaccharide 4 (identified as peak 4) was found to vary in concentration as a function of leaf position (Figure 3). Visual inspection of the trace (Figure 3) and quantitative analysis of peak areas (Figure 4A) showed that the arabinoxyloglucan oligosaccharide 4 subunit increased from leaf 3 to 5, but decreased in leaves 5 and 6, implying this arabinoxyloglucan component is under developmental control during leaf growth.

Given the importance of the arabinoxyloglucan component to the VvPGIP1 tobacco phenotypes (as suggested in [24]), enzymatic oligosaccharide profiling was performed on AIR sourced from all developmental stages (i.e. leaf positions 3–6) from both wild type and transgenic lines. Six major oligosaccharides were generated from all of the plant populations assayed and these were quantified using high performance anion exchange chromatography coupled to pulsed amperometric detection (Figure 4). Significant differences were found for oligosaccharide 4 (i.e. peak 4) abundance in wild type (Figure 4A) versus transgenic leaves (Figures 4B and C), less marked but significant differences were also found for peaks 1 and 2. Leaves 3, 4 and 5 from transgenic lines were consistently found to have substantially less of this arabinoxyloglucan oligosaccharide component than wild type leaves (Figure 4). The developmental trend/pattern however was unchanged between control and VvPGIP1 lines for leaves 3–5, supporting the position that VvPGIP1 does not influence normal growth and development (also supported by the fact that no visual phenotypic alterations are observed in the PGIP overexpressing lines). In the case of leaf 6 (oldest harvested leaf), the abundance of oligosaccharide 4 in the polymer has returned to near wild-type levels and no statistically significant differences were found (Figure 4). The most marked differences in abundance between wild-type and transgenic arabinoxyloglucan-derived oligosaccharide 4 was found for leaf position 3 (Figure 4D); all further detailed polymer fractionation and characterization presented are therefore only for this leaf position.

### Fractionation of AIR and comparative profiling of arabinoxyloglucan polymers sourced from wild- type and transgenic lines at leaf position 3

**Figure 5:**
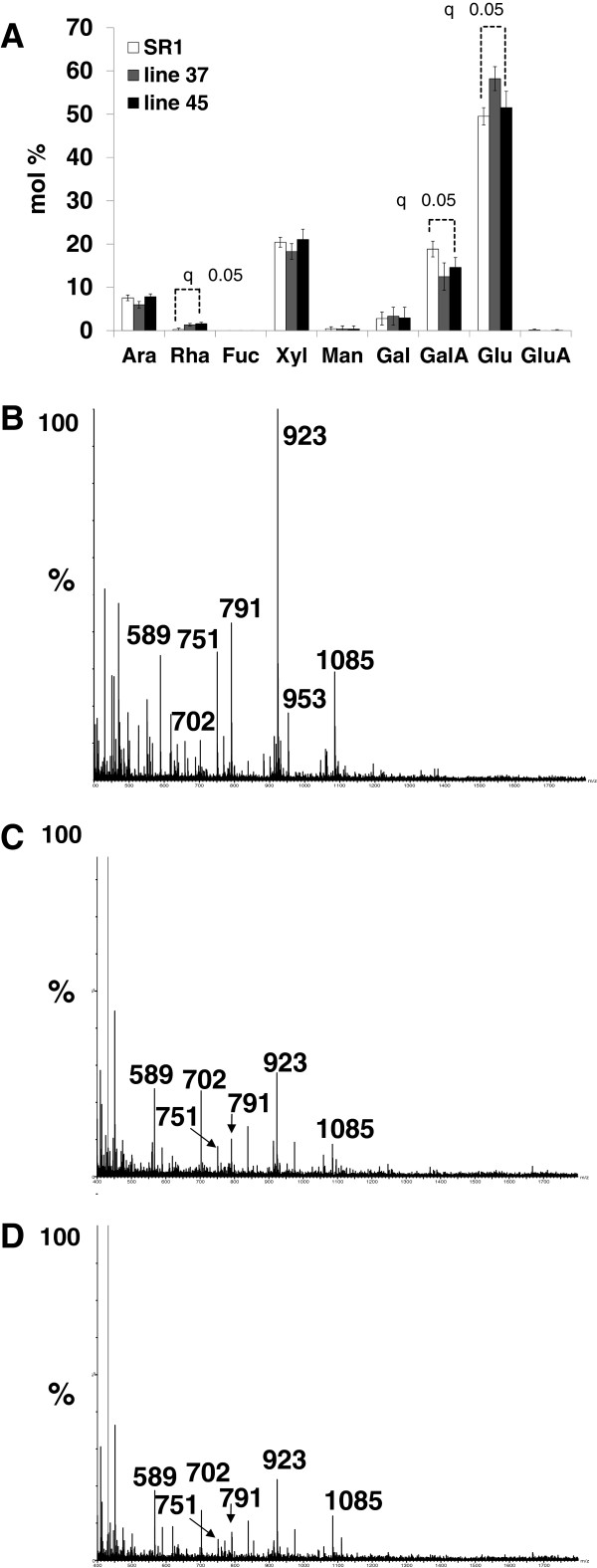
**Monosaccharide composition analysis of the arabinoxyloglucan-enriched fraction (prepared using ePGase and XEGase) isolated from ‘leaf 3-labelled’ leaves on SR1, 37 and 45 tobacco plants (A).** Electrospray Ionisation (ESI) mass spectrometric (MS) traces of oligomeric digestion products released from the arabinoxyloglucan-enriched fraction prepared from ‘leaf 3-labelled’ leaves on SR1, 37 and 45 tobacco plants (**B**). Analyses were performed using four biological replicates for the SR-1 (control) plants and for each of the transgenic plants. Two technical replicates were performed for each analysis.

To determine if changes to the global hemicellulose content had occurred in the transgenic lines a standard chemical fractionation protocol was employed on AIR, followed by gravimetric analysis. Apart from the 4 M KOH fractionation (rich in arabinoxyloglucan polymers) which appeared to show a small but statistically significant increase in abundance in transgenic versus wild type leaves (Additional file 3A) no major differences were found. These slight differences are difficult to interpret and may indicate a shift in polymer solubility between the different lines tested. Subsequent monosaccharide compositional analysis of each fraction (hot buffer- (A), sodium carbonate- (B) and 4 M KOH (C) soluble fractions) showed no major differences between samples (Additional file 4). Profiling of the 4 M KOH soluble fraction using PspXEG5 showed no compositional differences between the different plant populations (data not shown). As it is known from literature that plant cell walls are composed of enzyme ‘accessible’ and ‘inaccessible’ domains [31], the use of chemical extractants which indiscriminately degrade polymers may result in datasets difficult to interpret in terms of enzyme-mediated processes (such as XET activity/action). To address this, an enzymatic fractionation protocol, using ePG and PspXEG5 treatments, was also used on tobacco AIR sourced from leaf 3 samples (as outlined and performed by Nguema-Ona et al. [26]). Gravimetric analysis (Additional file 3B) showed broadly similar results to that obtained using chemical treatment. Analysis of the XEG-soluble material using monosaccharide compositional analysis revealed shifts in the GalA and Glc content possibly indicating polymer solubility changes in this domain between lines (Figure 5A). Further compositional analysis was performed using enzymatic oligosaccharide mass spectrometry.

The PspXEG5 facilitated arabinoxyloglucan-derived oligosaccharide mixtures (previously obtained from leaf 3) were directly injected into a high resolution mass spectrometer and analysed by ESI-MS in positive mode (Figure 5B-D). Almost all the ions found in the spectra were common to both the transformed and wild type lines; including m/z 791, 923 and 1085; which correspond to Hex_3_Pen_2_ (XXG/ SGG), Hex_3_Pen_3_ (SXG, XSG) Hex_4_Pen_3_ (SXGG, XSGG) oligomers respectively, identified in Nguema-Ona et al. [26]. A relative decrease in abundance of the m/z 923 ion was observed in the transformed lines when compared to the neighboring ions m/z 791 and 1085. This appears to correlate with the decrease of the peak 4 oligosaccharide (analyzed by HPAEC-PAD) previously reported in this study (see Figure 4, and also Additional file 5 where the HPAEC-PAD data from leaf 3, presented in Figure 4, is directly compared to the transgenic lines). Further work is necessary to elucidate the identity of these components more fully which is beyond the scope of this current profiling study. Finally, PspXEG5-insoluble material was further extracted with 4 M KOH, neutralized, dialyzed, freeze-dried and suspended in sodium acetate buffer, before being digested with PspXEG5. The addition of PspXEG5 is necessary to liberate all exposed, i.e. less tightly bound xyloglucan chains, and thereafter KOH is added to compete with the strong hydrogen bonding of the xyloglucan component tightly-bound to cellulose microfibrils, as performed in Ngeuma-Ona et al.[26], the released chains are then susceptible to enzyme action. No differences were found in released oligosaccharide patterns between wild type and transgenic lines using ESI-MS (Additional file 6) or HPAEC-PAD (Additional file 7), similarly for total monosaccharide composition of released material (Additional file 8), for the XEG-insoluble fraction.

## Discussion

*V. vinifera* polygalacturonase-inhibiting protein 1 (VvPGIP1) is active against fungal ePGs and constitutive expression of the encoding gene led to tobacco plants with decreased susceptibility against *B. cinerea*[16,23]. When these plants were subjected to gene expression and biochemical analyses in the absence of infection, important changes to cell wall modulating activities, particularly the xyloglucan endotransglycosylase/hydrolases (XTHs) were discovered [24]. Here we investigated the cell walls of the VvPGIP1 tobacco population further by using high-throughput and fractionation techniques optimized for tobacco leaves [26].

### Vvpgip1 overexpression results in subtle remodeling of tobacco leaf cell walls and alters the accessibility of the arabinoxyloglucan-cellulose network without affecting general cell wall biosynthesis and turnover

The CoMPP datasets did not point to wholesale biosynthetic compositional changes in the transgenic lines (Figure 1). This was also borne out by the later cell wall compositional profiling datasets (Figures 2, 3, 4). Cell wall profiling of the untransformed control leaves provided valuable baseline data on leaf cell wall development and turnover (as a function of leaf age) for ultimate comparison with transgenic lines. The developmental pattern of pectin polymers (i.e. Gal and GalA levels) observed in the leaves resembled pectin maturation in the wall. Pectin maturation during deposition/turnover is known to involve a shift from methylesterified pectin HGA to the de-esterified form and linked to a parallel rigidification in the tissue as calcium ‘egg box’ cross-linking occurs [37]. Labavitch and Ray [29] and later, Nishitani and Masuda [30] commented on a turnover of galactose-containing cell wall polysaccharides in elongating pea stem segments and growing azuki bean epicotyls respectively. Various studies have shown that β-1,4-galactan associated to RG-I pectin is regulated in relation to cell expansion [38,39]; it is also known to accumulate at the onset of cell elongation [40], and is progressively degraded during and after the elongation/expansion phase [41]. Similarly, our data indicated that during tobacco leaf maturation the Gal content (possibly representing substituted galactans linked to RG-1) decreased and the free unsubstituted pectin HGA content increased which can then undergo calcium ion binding and rigidification. This developmental pattern for Gal and GalA levels (i.e. pectin) was not altered in the transgenic lines.

The modulation of the xyloglucan composition of the VvPGIP1 transgenics reported in this study, together with the data presented by Alexandersson et al. [24] suggests that a reorganization of the cellulose-xyloglucan network has taken place that favours “strengthening”. This is supported by the CoMPP data for particularly mAb LM15 and to a lesser extent mAb CBM3a, where decreased accessibility to these mAbs was observed in the transgenic lines. The CoMPP datasets also showed subtle remodeling occurring, as reflected by the binding of mAbs JIM7, JIM8 and JIM13. The enhanced binding observed for mAb JIM7 suggested increased esterification of pectin in transgenic lines, which would require further confirmatory investigations on the pectic components of the transgenic lines. Pectin methyl esterification has been proposed to play a role in plant-pathogen interactions principally by limiting the accessibility of pectin to degradation by fungal enzymes [42]. Expression of pectin methyl esterase inhibitor proteins (PMEIs) by plants is believed to block/alter the action of native pectin methyl esterases (PMEs) *in planta* thereby modulating the level of pectin esterification [42-44]. The level of pectin methyl esterification, mediated by expression of PMEIs, in turn has been correlated with a reduction in infection of *Botrytis cinerea*[44]. It is certainly possible that elevated levels of pectin esterification in our transgenic lines, in conjunction with the xyloglucan-cellulose modifications, are responsible for the resistance phenotypes observed during trial infections. Currently there is insufficient data on pectin (in general) and esterification levels (in particular) to support this conclusion and further experiments are underway to perform detailed analysis of pectin changes in transgenic leaves to validate the CoMPP datatsets reported in this study.

### VvPGIP1 overexpression most likely leads to alterations in the arabinoxyloglucan XEG-soluble composition through *in muro* modification

The mechanical resistance of the plant cell wall is a factor important in plant-pathogen interactions as most pathogens need to disrupt wall organization during infection [7]. This resistance depends on the integrity of the cellulose/hemicellulose network [31,45,46]. Pauly et al. [31] demonstrated that the xyloglucan network is organized into three distinct and structurally different domains: a xyloglucan-specific endoglucanase (XEG)-extractable domain (the XEG-soluble domain), a strong alkaline-extractable domain (XEG-insoluble domain), and finally a third domain entrapped within or between cellulose microfibrils. The XEG-soluble domain in VvPGIP1 transformed plants was found to be altered (Figure 4) and this is likely to have occurred *in mur*o. Two types of processes involved in xyloglucan metabolism have been well documented: The first involves a change in the biosynthesis of xyloglucan [47], and the second an alteration in the *in muro* metabolism of xyloglucan [48]. In our case changes in xyloglucan biosynthesis are unlikely and no evidence has been found for this (Figures 2, 3, 4). Mutations in fundamental biosynthetic pathway genes have been studied and reported to lead to differences in xyloglucan polymer composition in all three domains; this type of wholesale modification also tends to produce altered morphological and/or growth phenotypes [36,49,50], which is not what was observed in the plants studied here. Hence, *in muro* changes are a more probable explanation for the alterations that were observed in the *Vvpgip1* overexpressing lines (Figures 4 and 5). This type of modification has been shown to be restricted to the XEG-soluble domain of polymeric xyloglucan [32,33], since only cell wall modifying enzymes responsible for this *in muro* modification are able to access this domain. It was shown that the expression of some *XET/XTH* genes were downregulated in healthy VvPGIP1 tobacco plants which caused a lower total enzymatic activity of XET/XTH in these lines [24]. It is therefore highly probable that the compositional changes observed for the XEG-soluble domain in VvPGIP1 transformed tobacco lines are a result of modified *in muro* enzymatic activity (i.e. remodeling) and did not occur at the Golgi body.

It is reasonable to think that the changes to the XET/XTH enzymatic activity, in conjunction with the action of the other cell wall apoplastic enzymes acting on the XEG-soluble fraction promoted the modification we observed in the oligosaccharide composition of the XEG-soluble fraction (Figure 4). A number of apoplastic enzymes work in concert and act on the XEG-soluble fraction and have the potential to modify xyloglucan polymers. Endoglucanases, XTHs having XET activity [51], α-xylosidases [52], β-galactosidases [53], and α-fucosidases [54] can contribute to the final polymer composition in the XEG-soluble domain. Xyloglucan modifications *in muro* have been well studied in *Arabidopsi*s and mutants with changes in glycosidase activity, such as the Atxyl1, Atbgal10 and axy8 mutants [52-54] show significant alterations in the composition of the XEG-soluble polymers. It has recently been shown that trans-α-xylosidases and trans-β-galactosidases can act directly on the xyloglucan polysaccharide to cause modification [55].

Sampedro et al. [52] showed that in addition to the modification which occurred in the polymeric xyloglucan of the AtXyl1 mutant, two intermediates of xyloglucan metabolism (XXXG and XXLG) substantially accumulated in the growth media and probably in the soil around the roots. Such accumulation of apoplastic, free and diffusible xyloglucan oligosaccharides were not found in the Atbgal10 and axy8 mutants, respectively deficient in a β-galactosidase and an α-fucosidase involved in the modification of xyloglucan *in muro*. It thus would be of interest to investigate the occurrence of apoplastic xyloglucan oligosaccharides in our transformed lines, given the modifications observed in the xyloglucan fraction.

### A proposed model for the effect of Vvpgip1 overexpression on cell wall architecture

**Figure 6:**
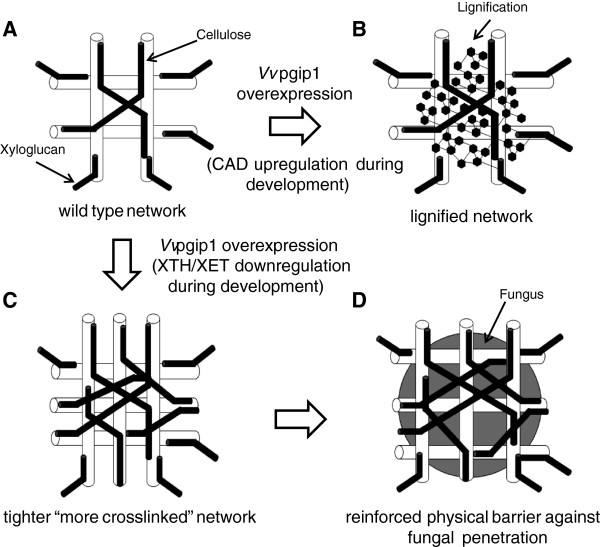
**A proposed model summarizing the influence of VvPGIP1 overexpression on tobacco leaf cell wall-associated processes and also suggesting the effect of these processes on the cell wall molecular architecture/functioning during pathogen infection.** VvPGIP1 overexpression leads to CAD upregulation and lignification (**A** to **B**). VvPGIP1 overexpression leads to XTH/XET downregulation (transcript activity and enzyme activity) in tobacco leaves resulting in a more tightly-bound/strengthened cellulose-hemicellulose network (**A** to **C**). In the presence of a pathogenic fungus, such as *Botrytis cinerea*, this modified cellulose-hemicellulose network acts as a ‘reinforced’ physical barrier to penetration (**C** to **D**).

Fungal strains are able to effectively degrade pectin rich matrics, but can be slowed-down in their attempt to physically penetrate and macerate a reinforced cellulose-xyloglucan network [7]. The recent sequencing of the *B. cinerea* genome showed that its arsenal of cell wall degrading enzymes (CWDEs) is very limited against hemicelluloses such as xyloglucan [56]. With the additional analyses provided in this study it is now possible to produce a working hypothesis (Figure 6) summarizing the known effects of VvPGIP1 overexpression on cell wall-associated processes and –architecture in tobacco (*N. tabacum*). VvPGIP1 overexpression is able to induce lignin accumulation (Figure 6A-B; [24]), pectin modification (mAb JIM7 binding; this requires further confirmation) and arabinoxyloglucan-cellulose re-organization (Figure 6C, D and E; CoMPP analysis, arabinoxyloglucan analysis and [24]). The clear consensus from this study is that VvPGIP1 overexpression is able to cause modification and re-organization that result in a general strengthening/reinforcing of the cell wall in advance of infection. Once infection occurs, the PGIPs would form inhibition complexes with the ePG, while the altered (strengthened) wall matrix further functions to impede penetration of the fungus.

## Conclusion

Our results show that the wall developmental profile was altered in the transgenic VvPGIP1 lines versus the wild-type controls. We also showed that polysaccharides of the XEG-soluble domain were modified resulting in an alteration of the enzyme-derived oligosaccharide profiles obtained from transgenic material. These changes did not significantly influence plant morphology or normal growth processes compared to wild-type lines. VvPGIP1 overexpression therefore results in cell wall remodeling and reorganization of the cellulose-xyloglucan network in tobacco in advance of potential infection and provides further proof of a possible priming effect caused by the overexpression of the VvPGIP1 and a non-ePG-related function for PGIPs *in vivo*.

It is possible that PGIPs evolved from precursor genes that had roles outside of plant defence and that during the course of evolution were co-opted as defence proteins because of their unique properties (i.e. protein and pectin binding). This also suggests that *pgip* genes probably have a number of other functional roles *in planta*, as a consequence of their evolution, including participation in plant signaling mechanisms. Our data adds to the growing list of alternative functions identified for PGIPs lately and show that VvPGIP1 play a role in modifying xyloglucan composition *in muro* prior to infection. The functional role for VvPGIP1 in grapevine, its native genetic background, is now clearly of interest. *Vvpgip1* is predominantly expressed in berries, with expression peaking at véraison (at the onset of ripening) and to a lesser extent in roots (Joubert et al., 2012) under normal (healthy) conditions. *Vvpgip1* expression is inducible by infection and wounding (in all organs) as well as hormone treatments and osmotic stress [22]. Berry ripening involves substantial sugar accumulation among other substances and this process starts at the onset of ripening. It is therefore possible that given the observed hemicellulose-cellulose network and the remodeling properties of VvPGIP1 (this study) that this protein has a role in osmotic regulation in berries by tightening/restricting the xyloglucan load-bearing matrix to cope with the developmental changes in the ripening berry. Unraveling the *in planta* functional roles of VvPGIP1 in relation to plant-pathogen interactions and berry development remain a high priority and are supported by this approach to subject transgenic phenotypes to various profiling technologies which has proved valuable in deciphering the *in planta* functions of PGIPs.

## Methods

### Generation of transformed tobacco lines constitutively expressing VvPGIP1

The generation of the transformed tobacco lines 37 and 45 (in the back ground *Nicotiana tabacum* (L. Petite Havana SR-1) constitutively expressing *Vitis vinifera pgip1* (*Vvpgip1)* and their phenotypic and genetic characterization were previously described in Joubert et al. [16,23] and Alexandersson et al. [24].

### Plant growth and harvesting conditions

Tobacco SR-1 as WT control and two transgenic lines (line 37 and line 45) were used in this study. Growth conditions were as described in [26]. Briefly, control and transgenic tobacco seeds were sown on solid MS media [57] in Petri dishes and incubated at 26°C with a 16 hrs light / 8 hrs dark photoperiod regime. Seedlings were transferred to soil and grown in a climate room (23°C; 16 hrs light 8 hrs dark photoperiod) until fully mature plants (8 leaf stage) were available. Four fully expanded leaves (numbered leaf 3 for the younger leaf to leaf 6 for the older one) were harvested separately per plant and flash-frozen using liquid nitrogen and stored at -80°C until further use. In order to yield a reasonable amount of alcohol insoluble residue (AIR) per sample, three individual leaves, from three individual plants of the same line (and the same leaf position) were pooled together after the harvesting, flash-frozen together and counted as one biological sample. For statistical purposes, four to eight biological samples were collected per leaf position (leaf position 3 to leaf position 6) and per line (control SR-1, VvPGIP1 line 37 and VvPGIP1 line 45).

### Cell wall isolation and fractionation

Cell wall materials were extracted from frozen tobacco leaves and fractionated according to [26]. Briefly, 2 grams of frozen leaves were ground, under liquid nitrogen using a mortar and pestle, to a fine powder. After boiling in 80% ethanol for 20 min, insoluble material was washed in methanol: chloroform (1:1) for 24 hrs, and this was performed with fresh solvents for an additional 24 h due to the high lipid/oil content, thereafter the residue was washed in methanol before air drying. The dry material, referred to as alcohol insoluble residue (AIR), was de-starched using a combination of thermostable α-amylase, amyloglucosidase and pullulanase (all from Megazyme). De-starched AIR was independently, both chemically and enzymatically fractionated.

### Carbohydrate microarray polymer profiling (CoMPP) analysis of cell wall material

AIR was prepared from frozen tobacco leaves as described in the ‘cell wall isolation and fractionation’ section of this paper. For the CoMPP analysis, the chemical fractionation process was simplified to three extraction steps. CoMPP analysis was performed by extracting with CDTA (to predominantly extract pectin-type material) and NaOH (to predominantly extract hemicellulosic polymers) from AIR. Finally a cadoxen extraction was applied which facilitated the partial solubilization of the remaining cellulose-rich residue. Approximatively 40 mg of material was used to perform the CoMPP analyses as described in [27]. After weighing approximately 10 mg for each sample the extraction volume is adjusted accordingly to a ratio of 30 μl extraction solution per mg of AIR. The values in the heatmap are mean spot signals from three experiments and the highest signal in the entire data set was set to 100 and all other data adjusted accordingly. A cut off value of 10 was imposed.

### Monosaccharide composition analysis by gas chromatography

A gas liquid chromatography method [58] was used to determine the monosaccharide content of cell wall residues and fractions. Approximately 1–3 mg of wall residue or fractionated material was hydrolyzed (2 M TFA, 110°C, 2 h) and the liberated monosaccharides converted to methoxy sugars using 1 M methanolic HCl at 80°C for 24 h. Silylation was performed at 80°C (20 min) to produce trimethyl-silyl-glycosides which were dissolved in cyclohexane. The derivatives were separated and analyzed in a gas chromatograph (Hewlett Packard 5890 series II) coupled to a flame ionization detector, using a 30 m × 0.25 mm (i.d.) HPS-MS column. The oven temperature program was stabilized at 120°C for 2 min, ramped at 10°C/min to 160°C, then at 1.5°C/min to 220°C and finally at 20°C/min to 280°C. Myo-inositol (0.5 μmol) was used as the internal standard. Derivatives were identified based on their retention time and quantified by determination of their peak areas. Monosaccharides (from Sigma-Aldrich) were used as standards to determine the retention time of the nine main monosaccharides found in plant cell walls. The sugar composition was expressed as mole percentage of each monosaccharide. Error bars in the histograms represent the standard deviation (SD) of the mean of four biological samples with two technical replicates per biological sample.

### High performance anion exchange chromatography

XEG-generated arabinoxyloglucan oligosaccharides were analyzed by high performance anion exchange (HPAE) chromatography (Dionex Ultimate 3000) equipped with a CarboPac PA-1 column combined with pulsed amperometric detection (PAD; Coulochem 111 detector). Samples (10 μL) were eluted at 1 mL/min with the following sodium acetate gradient in 100 mM NaOH: 0–2.5 min: 100 mM NaOH, 2.5–3 min: linear gradient of 0–70 mM NaOAc, 3–29 min: linear gradient of 70–100 mM NaOAc, 29–29.1 min: linear gradient 100–500 mM NaOAc, 29.1–38 min, 500 mM NaOAc, 38–47 min, 100 mM NaOH.

### Gravimetric analysis of chemically and sequentially extracted de-starched AIR

Three chemically and sequentially extracted fractions (50 mM acetate sodium hot buffer, 50 mM CDTA, 4 M KOH; also see [26]) were dialyzed (3.5 kDa cutoff dialysis tubing) against deionized water (48 hrs at 8°C), freeze-dried before gravimetric and compositional analyses (see below) were conducted. The 4 M KOH soluble fraction was also digested with XEGs to obtain an XEG-soluble fraction for compositional analysis. For a full description of the fractionation process and the polysaccharides extracted using the methodology refer to Nguema-Ona et al. [26].

### Gravimetric analysis of enzymatically and sequentially extracted de-starched AIR

In order to perform a gravimetric analysis of tobacco arabinoxyloglucans (adapted from [31]), AIR was processed as follows: 50 mg of AIR was initially treated twice with an ePG (GH28 from *Aspergillus* sp; Megazyme) for 18 hours at 37°C (2 mg/U), first in 50 mM AcNA buffer pH 4, followed by a second treatment in 50 mM ammonium acetate, 50 mM CDTA and 0.5% NaN_3_ (see also [59]). After collection of the soluble fraction, the residue was washed with deionized water, treated for 20 min at 95°C in a water-bath in order to solubilise residual pectin material and to inactivate ePG activities, and then washed twice with deionized water. The amount of solubilized pectin was deduced from the weight of dry residual depectinated material. This resulting depectinated material was then treated with the PspXEG5 (18 hours at 37°C; 2 mg /U) to yield a PspXEG5 fraction PspXEG5 F1. Residual material obtained (XEG-insoluble material), was washed extensively with deionized water, freeze-dried and the residual dry mass was weighted out. The XEG insoluble material was extracted with 4 M KOH in order to release an XEG-insoluble hemicellulosic material enriched in arabinoxyloglucan partially associated to the cellulose *in muro* (see [31]). The remaining material was washed twice with deionized water and freeze dried. Prior to further analyses, ePG-soluble fractions were dialyzed (3.5 kDa cutoff dialysis tubing) against deionized water (48 hrs at 8°C), and freeze-dried. XEG-soluble fractions were either filtered (0.22 μm nylon membrane) prior to analysis by HPAE chromatography, or further desalted and concentrated as described by Packer et al. [60], by using a graphitized solid phase extraction (UltraClean SPE columns; Altech, USA) column and a Visiprep vacuum manifold (Supelco, USA). SPE columns were conditioned with 4 mL of 90% aqueous (v/v) acetonitrile in 0.1% aqueous (v/v) trifluoroacetic acid (TFA) and with 4 mL of distilled water. 1 mL of extract was applied to the equilibrated column and then washed with 4 mL of deionized water after adsorption. To elute oligosaccharides, the columns were rinsed with 4 mL of 25% aqueous (v/v) acetonitrile in 0.1% (v/v) TFA. The fractions were finally freeze-dried and re-suspended in deionized water before further analyses were performed using mass spectrometry and gas liquid chromatography.

### ESI-MS analyses of arabinoxyloglucan oligosaccharides

XEG-soluble arabinoxyloglucan oligosaccharides (generated after digestion of depectinated material by PspXEG5) and XEG-insoluble arabinoxyloglucan oligosaccharides (generated after the digestion of the XEG-insoluble arabinoxyloglucan by *Psp*XEG5) were desalted and concentrated as described in [26] and finally analyzed by electrospray ionisation mass spectrometry (ESI-MS) using a Synapt G2 high definition MS. Samples (1 μL) were directly injected into a stream of 80% acetonitrile, 0.1% formic acid using a Waters UPLC at flow rate of 0.2 mL/min. The ion source was set as follows: Source Electrospray positive; Capillary voltage 3 kV; Cone Voltage 40 V. Leucin enkaphalin was used as the lock mass. The mass spectrometer was calibrated with sodium formate. In order to identify the possible structure corresponding to major ions detected, an accurate mass determination was performed using the MassLink software. We deduced the partial chemical structures of released oligosaccharides by comparison with existing literature [26,59,61,62].

### Statistical analyses

Descriptive statistical analyses and analysis of the variance (One way ANOVA) were performed with the statistical package of Microsoft Excel 2010 and Statistica 10 software. All the test were performed at p = 0.05. Significant differences are indicated in the Figures and Additional Files.

## Abbreviations

AIR: Alcohol insoluble residue; AXyG: Arabinoxyloglucan; CBM: Carbohydrate binding module; CoMPP: Comprehensive microarray polymer profiling; ePG: Endopolygalacturonase; ESI-MS: Electrospray ionization – mass spectrometry; HG: Homogalacturonan; HPAEC-PAD: High performance anion exchange chromatography – pulse amperometric detection; mAb: Monoclonal antibody; OGA: Oligogalacturonan; PspXEG5: *Paenibacillus sp* xyloglucan specific endoglucanase 5; RG: Rhamnogalacturonan; RT-PCR: Real time – polymerase chain reaction; XEG: Xyloglucan-specific endoglucanase; XyG: Xyloglucan

## Competing interests

The authors declare that they have no competing interests.

## Authors’ contributions

MAV, JPM and ENO conceptualized the study and were involved in the experimental layout. ENO did the sampling, processing of samples and subsequent analyses. AH provided expert advice in analysis on the HPAEC-PAD. AF, JUF and WGTW processed the samples for COMPP analysis and helped with interpretation of the data and preparation of the relevant figure. JPM prepared Figure 6. ENO, JPM and MAV drafted the initial and final manuscript. All authors contributed to discussion of the results, reviewing of the manuscript and approved the final manuscript.

## Supplementary Material

Additional file 1 **Evaluation of the leaf developmental cell wall composition profile of VvPGIP1 (lines 37) tobacco plants.** Monosaccharide composition analysis of (A) the destarched AIR material, (B) polymers released after endopolygalacturonase (ePGase) and (C) subsequent xyloglucanase (XEGase) treatment of alcohol insoluble residue (AIR) prepared from VvPGIP1 transformed line 37 tobacco leaves (individual leaves analysed and labelled leaf 3 to leaf 6 are indicated). Ara: arabinose; Rha: rhamnose; Fuc: fucose; Xyl: xylose; Man: mannose; Gal: galactose; Glu: glucose; GalA: galacturonic acid; GluA: glucuronic acid. Analyses were performed using four biological replicates for the SR-1 (control) plants and for each of the transgenic plants. Two technical replicates were performed for each analysis. Click here for file

Additional file 2 **Evaluation of the leaf developmental cell wall composition profile of VvPGIP1 (lines 45) tobacco plants.** Monosaccharide composition analysis of (A) the destarched AIR material, (B) polymers released after endopolygalacturonase (ePGase) and (C) subsequent xyloglucanase (XEGase) treatment of alcohol insoluble residue (AIR) prepared from VvPGIP1 transformed line 45 tobacco leaves (individual leaves analysed and labelled leaf 3 to leaf 6 are indicated). Ara: arabinose; Rha: rhamnose; Fuc: fucose; Xyl: xylose; Man: mannose; Gal: galactose; Glu: glucose; GalA: galacturonic acid; GluA: glucuronic acid. Analyses were performed using four biological replicates for the SR-1 (control) plants and for each of the transgenic plants. Two technical replicates were performed for each analysis. Click here for file

Additional file 3 **Gravimetric analysis of AIR fractionated using chemical (A) and enzymatic (B) methods prepared from SR-1 and transgenic (lines 37 and 45) tobacco leaf-3 labeled leaves.** Refer to Nguema-Ona et al. [26] (Figure 2) for an outline of fractionation procedure used. Analyses were performed using four biological replicates for the the SR-1 (control) plants and for each of the transgenic plants. Two technical replicates were performed for each analysis. Click here for file

Additional file 4 **Monosaccharide composition analysis of the (A) hot buffer-, (B) sodium carbonate and (C) 4 M KOH- soluble fractions, prepared from SR-1 and transgenic (lines 37 and 45) tobacco leaves (at the leaf 3 position) and presented in additional file 3A.** Ara: arabinose; Rha: rhamnose; Fuc: fucose; Xyl: xylose; Man: mannose; Gal: galactose; Glu: glucose; GalA: galacturonic acid; GluA: glucuronic acid. Analyses were performed using four biological replicates for the the SR-1 (control) plants and for each of the transgenic plants. Two technical replicates were performed for each analysis. Click here for file

Additional file 5 **Relative abundance of peaks labelled (paid special attention to peaks labelled 1, 4 and 5) showing arabinoxyloglucan subunit compositions, present in chromatograph traces of oligomeric digestion products of de-pectinated AIR prepared from leaf 3, and sourced from SR1, line 37 and line 45 tobacco plants.** Analyses were performed using four biological replicates for the the SR-1 (control) plants and for each of the transgenic plants. Two technical replicates were performed for each analysis. Click here for file

Additional file 6 **Electrospary ionisation mass spectrometric (ESI-MS) data of the XEG-insoluble fractions prepared from SR-1(A) and VvPGIP1 line 37 (B) and 45 (C) tobacco leaves.** Analyses were performed using four biological replicates for the SR-1 (control) plants and for each of the transgenic plants. Two technical replicates were performed for each analysis. Click here for file

Additional file 7 **Liquid chromatographic (HPAEC-PAD data of the XEG-insoluble fractions prepared from SR-1(A) and VvPGIP1 line 37 (B) and 45 (C) tobacco leaves.** Analyses were performed using four biological replicates for the SR-1 (control) plants and for each of the transgenic plants. Two technical replicates were performed for each analysis. Click here for file

Additional file 8 ** arabinose; Rha: rhamnose; Fuc: fucose; Xyl: xylose; Man: mannose; Gal: galactose; Glu: glucose; GalA: galacturonic acid; GluA: glucuronic acid.** Analyses were performed using four biological replicates for the SR-1 (control) plants and for each of the transgenic plants. Two technical replicates were performed for each analysis. Click here for file
