# Supplementary material for: Overexpression of the grapevine PGIP1 in tobacco results in compositional changes in the leaf arabinoxyloglucan network in the absence of fungal infection
Source: BMC Plant Biol. 2013 Mar 18;13:46. doi: 10.1186/1471-2229-13-46 (PMC3621556; doi:10.1186/1471-2229-13-46)
Supplement: Additional file 1 — Evaluation of the leaf developmental cell wall composition profile of VvPGIP1 (lines 37) tobacco plants. Monosaccharide composition analysis of (A) the destarched AIR material, (B) polymers released after endopolygalacturonase (ePGase) and (C) subsequent xyloglucanase (XEGase) treatment of alcohol insoluble residue (AIR) prepared from VvPGIP1 transformed line 37 tobacco leaves (individual leaves analysed and labelled leaf 3 to leaf 6 are indicated). Ara: arabinose; Rha: rhamnose; Fuc: fucose; Xyl: xylose; Man: mannose; Gal: galactose; Glu: glucose; GalA: galacturonic acid; GluA: glucuronic acid. Analyses were performed using four biological replicates for the SR-1 (control) plants and for each of the transgenic plants. Two technical replicates were performed for each analysis. [file 1471-2229-13-46-S1.pdf]

Additional File 1. Percent of Cell Wall Glycosyl -Residue Composition of VvPGIP1 tobacco transgenic leaves compared to the wild-type (see Figure 2).

			transgenic mol% / WT mol % x 100								
	transgenic lines	leaf position[b]	**Ara**	**Rha**	**Fuc**	**Xyl**	**Man**	**Gal**	**Glc**	**GalA**	**GlcA**
	VvPGIP1 line 37	**L3**	110,98	104,17	87,20	99,13	93,33	94,71		102,13	86,63
	VvPGIP1 line 37	**L4**	107,45	105,48	52,27	92,59	106,70	99,83		99,46	91,77
	VvPGIP1 line 37	**L5**	106,28	98,35	63,45	111,25	109,85	100,83		96,81	93,56
	VvPGIP1 line 37	**L6**	99,72	94,20	82,20	101,62	107,70	107,82		98,62	***83,85***
	VvPGIP1 line 45	**L3**	94,53	89,28	nd	87,40	113,32	116,03		92,79	***120,22***
	VvPGIP1 line 45	**L4**	110,68	97,81	63,73	98,77	116,15	113,46		91,44	85,14
	VvPGIP1 line 45	**L5**	77,71	80,74	29,52	87,04	***128,54***	***122,58***		98,24	104,88
AIR	VvPGIP1 line 45	**L6**	84,80	83,30	40,96	93,20	***124,68***	110,69		102,13	94,96
	VvPGIP1 line 37	**L3**	***84,15***	88,80	nd	***18,22***	***321,13***	***126,41***	100,35	***60,06***	***42,08***
	VvPGIP1 line 37	**L4**	112,01	***76,14***	***9,19***	***65,90***	nd	***139,54***	82,85	***212,94***	***75,19***
	VvPGIP1 line 37	**L5**	***80,82***	117,33	nd	***10,43***	***441,16***	116,14	103,98	***73,31***	***66,11***
	VvPGIP1 line 37	**L6**	84,75	***75,29***	nd	nd	***324,37***	***162,96***	95,55	***140,68***	***75,15***
	VvPGIP1 line 45	**L3**	109,93	***77,02***	**8,55**	***66,20***	***375,31***	***123,12***	90,35	109,81	***56,88***
	VvPGIP1 line 45	**L4**	***185,00***	***77,07***	**11,20**	108,89	nd	***136,68***	72,42	***275,45***	***69,84***
	VvPGIP1 line 45	**L5**	***132,95***	***68,52***	**24,27**	nd	***245,67***	***134,06***	95,54	***127,55***	105,52
EPG	VvPGIP1 line 45	**L6**	***196,13***	***86,56***	nd	***123,71***	***469,79***	***135,82***	87,90	***172,95***	88,62
	VvPGIP1 line 37	**L3**	92,85	***35,43***	nd	***79,88***	nd	***131,65***	124,65	137,30	nd
	VvPGIP1 line 37	**L4**	77,58	87,82	nd	87,38	nd	96,31	104,62	119,35	nd
	VvPGIP1 line 37	**L5**	70,94	***60,68***	nd	92,07	nd	***547,19***	102,96	127,31	nd
	VvPGIP1 line 37	**L6**	95,96	***69,44***	nd	108,89	nd	***259,93***	***70,87***	114,01	nd
	VvPGIP1 line 45	**L3**	92,24	***65,77***	nd	87,58	nd	96,18	140,65	121,20	nd
	VvPGIP1 line 45	**L4**	***77,35***	***68,99***	nd	105,21	nd	***54,45***	101,01	116,11	nd
	VvPGIP1 line 45	**L5**	***79,72***	***59,70***	nd	109,23	nd	***145,09***	114,22	115,59	nd
XEG	VvPGIP1 line 45	**L6**	***81,66***	***68,26***	nd	99,06	nd	***260,46***	87,35	121,71	nd

a Data represent four independent TMS GC–MS reactions from 4 (transgenic leaves) and 8 (wild type) independent wall extractions. Residues are abbreviated according to Figure 3.

b The walls used for glycosyl residue analysis were harvested from leaf position 3 (L3), leaf position 4 (L4), leaf position 5 (L5) and leaf position 6 (L6).

c Bold highlighted italicized values indicate transgenic glycosyl residue compositions that were statistically and ± 15% different from the WT mean.
