# Supplementary figures and images for: Overexpression of the grapevine PGIP1 in tobacco results in compositional changes in the leaf arabinoxyloglucan network in the absence of fungal infection
Source: BMC Plant Biol. 2013 Mar 18;13:46. doi: 10.1186/1471-2229-13-46 (PMC3621556; doi:10.1186/1471-2229-13-46)

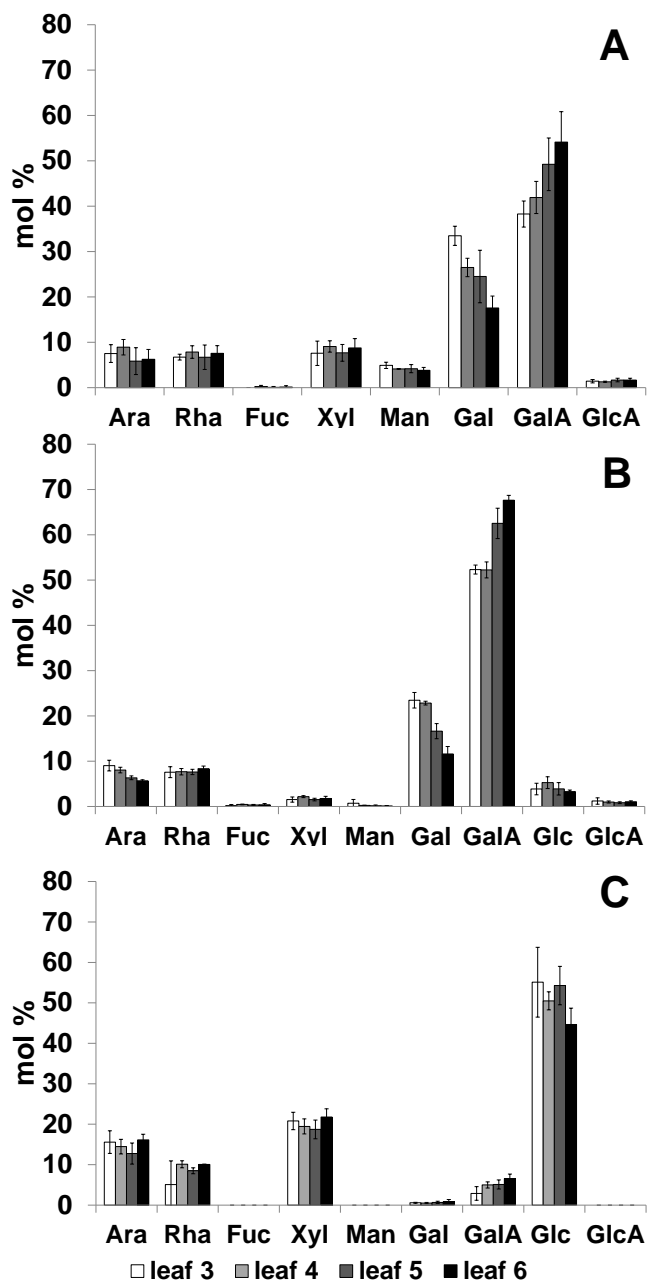

Additional file 2

Supplement: Additional file 2 — Evaluation of the leaf developmental cell wall composition profile of VvPGIP1 (lines 45) tobacco plants. Monosaccharide composition analysis of (A) the destarched AIR material, (B) polymers released after endopolygalacturonase (ePGase) and (C) subsequent xyloglucanase (XEGase) treatment of alcohol insoluble residue (AIR) prepared from VvPGIP1 transformed line 45 tobacco leaves (individual leaves analysed and labelled leaf 3 to leaf 6 are indicated). Ara: arabinose; Rha: rhamnose; Fuc: fucose; Xyl: xylose; Man: mannose; Gal: galactose; Glu: glucose; GalA: galacturonic acid; GluA: glucuronic acid. Analyses were performed using four biological replicates for the SR-1 (control) plants and for each of the transgenic plants. Two technical replicates were performed for each analysis. [file 1471-2229-13-46-S2.pdf]

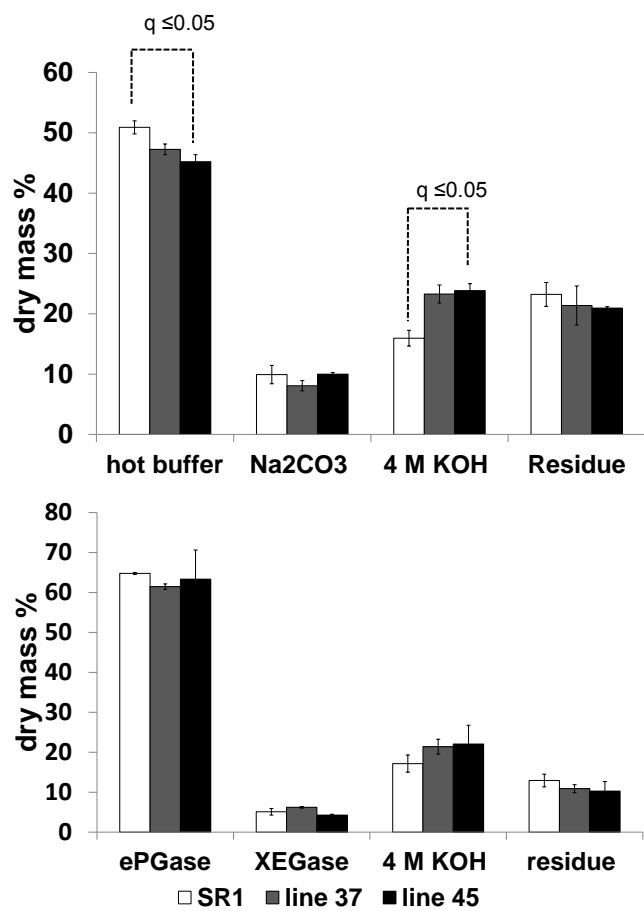

Additional file 3

Supplement: Additional file 3 — Gravimetric analysis of AIR fractionated using chemical (A) and enzymatic (B) methods prepared from SR-1 and transgenic (lines 37 and 45) tobacco leaf-3 labeled leaves. Refer to Nguema-Ona et al. [26] (Figure 2) for an outline of fractionation procedure used. Analyses were performed using four biological replicates for the the SR-1 (control) plants and for each of the transgenic plants. Two technical replicates were performed for each analysis. [file 1471-2229-13-46-S3.pdf]

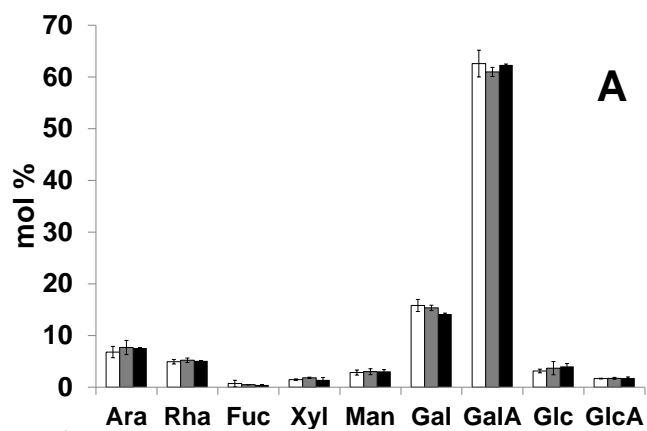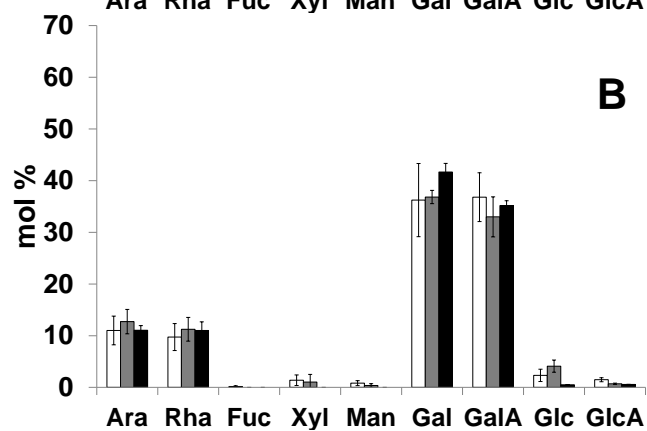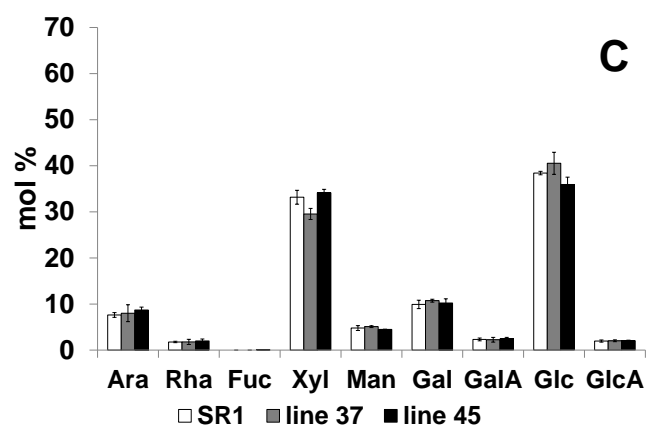

Additional file 4

Supplement: Additional file 4 — Monosaccharide composition analysis of the (A) hot buffer-, (B) sodium carbonate and (C) 4 M KOH- soluble fractions, prepared from SR-1 and transgenic (lines 37 and 45) tobacco leaves (at the leaf 3 position) and presented in additional file 3A. Ara: arabinose; Rha: rhamnose; Fuc: fucose; Xyl: xylose; Man: mannose; Gal: galactose; Glu: glucose; GalA: galacturonic acid; GluA: glucuronic acid. Analyses were performed using four biological replicates for the the SR-1 (control) plants and for each of the transgenic plants. Two technical replicates were performed for each analysis. [file 1471-2229-13-46-S4.pdf]

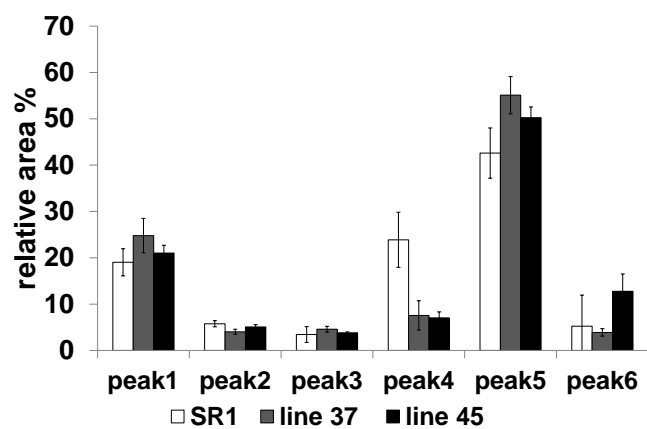

Additional file 5

Supplement: Additional file 5 — Relative abundance of peaks labelled (paid special attention to peaks labelled 1, 4 and 5) showing arabinoxyloglucan subunit compositions, present in chromatograph traces of oligomeric digestion products of de-pectinated AIR prepared from leaf 3, and sourced from SR1, line 37 and line 45 tobacco plants. Analyses were performed using four biological replicates for the the SR-1 (control) plants and for each of the transgenic plants. Two technical replicates were performed for each analysis. [file 1471-2229-13-46-S5.pdf]

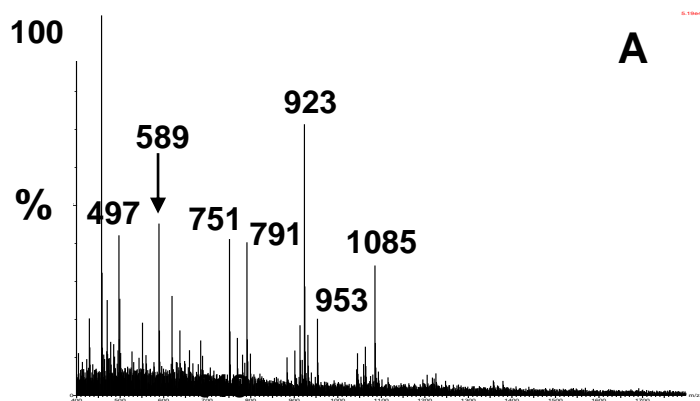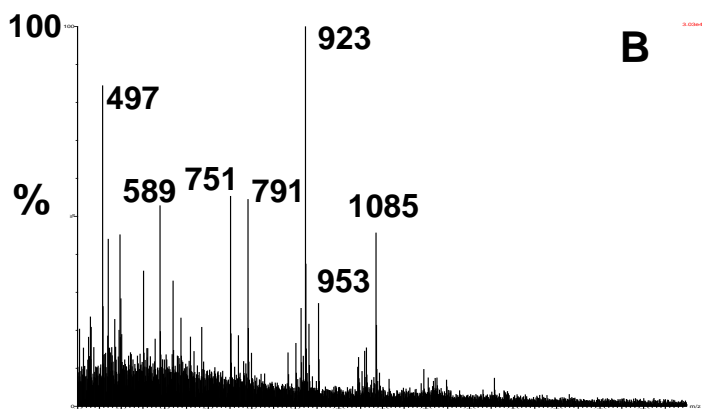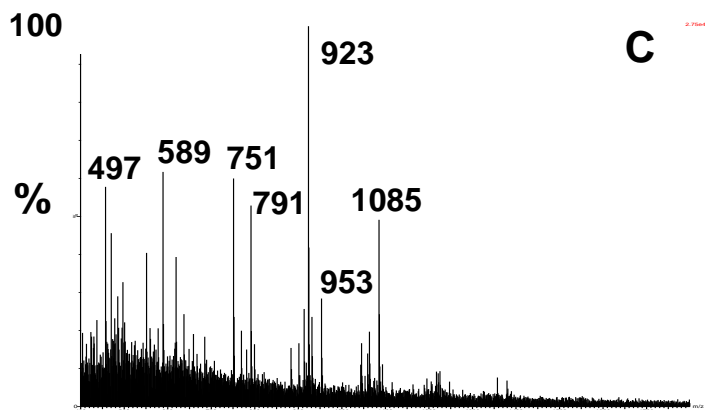

Additional file 6

Supplement: Additional file 6 — Electrospary ionisation mass spectrometric (ESI-MS) data of the XEG-insoluble fractions prepared from SR-1(A) and VvPGIP1 line 37 (B) and 45 (C) tobacco leaves. Analyses were performed using four biological replicates for the SR-1 (control) plants and for each of the transgenic plants. Two technical replicates were performed for each analysis. [file 1471-2229-13-46-S6.pdf]

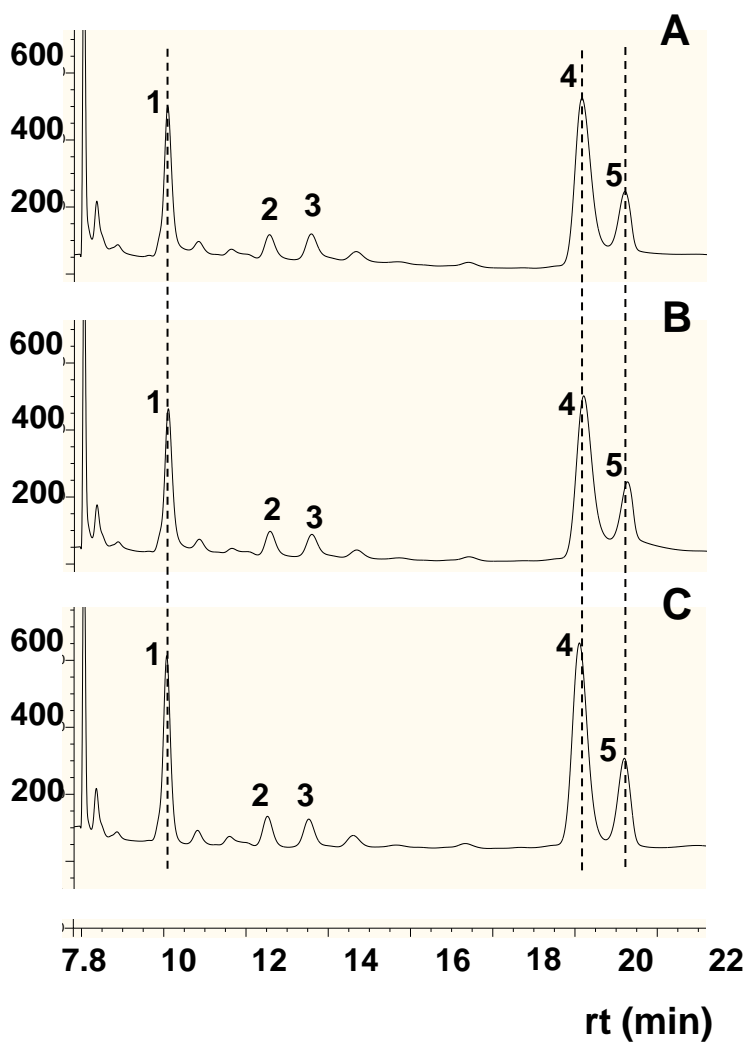

Additional file 7

Supplement: Additional file 7 — Liquid chromatographic (HPAEC-PAD data of the XEG-insoluble fractions prepared from SR-1(A) and VvPGIP1 line 37 (B) and 45 (C) tobacco leaves. Analyses were performed using four biological replicates for the SR-1 (control) plants and for each of the transgenic plants. Two technical replicates were performed for each analysis. [file 1471-2229-13-46-S7.pdf]

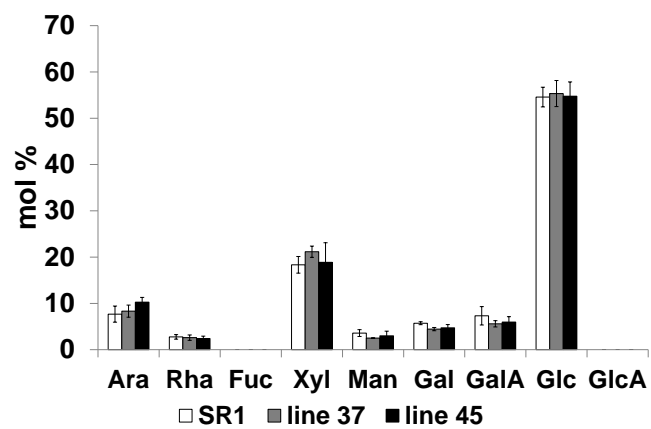

Additional file 8

Supplement: Additional file 8 — arabinose; Rha: rhamnose; Fuc: fucose; Xyl: xylose; Man: mannose; Gal: galactose; Glu: glucose; GalA: galacturonic acid; GluA: glucuronic acid. Analyses were performed using four biological replicates for the SR-1 (control) plants and for each of the transgenic plants. Two technical replicates were performed for each analysis. [file 1471-2229-13-46-S8.pdf]
